# Development of a Nomogram for Clinical Risk Prediction of Preterm Neonate Death in Ethiopia

**DOI:** 10.3389/fped.2022.877200

**Published:** 2022-05-27

**Authors:** Habtamu Shimels Hailemeskel, Sofonyas Abebaw Tiruneh

**Affiliations:** ^1^Department of Pediatrics and Neonatal Nursing, College of Health Sciences, Debre Tabor University, Debre Tabor, Ethiopia; ^2^Department of Public Health, College of Health Sciences, Debre Tabor University, Debre Tabor, Ethiopia

**Keywords:** preterm, death, prediction, nomogram, Ethiopia

## Abstract

**Introduction:**

In 2020, over 6,500 newborn deaths occured every day, resulting in 2.4 million children dying in their 1st month of life. Ethiopia is one of the countries that will need to step up their efforts and expedite progress to meet the 2030 sustainable development goal. Developing prediction models to forecast the mortality of preterm neonates could be valuable in low-resource settings with limited amenities, such as Ethiopia. Therefore, the study aims to develop a nomogram for clinical risk prediction of preterm neonate death in Ethiopia in 2021.

**Methods:**

A prospective follow-up study design was employed. The data were used to analyze using R-programming version 4.0.3 software. The least absolute shrinkage and selection operator (LASSO) regression is used for variable selection to be retained in the multivariable model. The model discrimination probability was checked using the ROC (AUROC) curve area. The model’s clinical and public health impact was assessed using decision curve analysis (DCA). A nomogram graphical presentation created an individualized prediction of preterm neonate risk of mortality.

**Results:**

The area under the receiver operating curve (AUROC) discerning power for five sets of prognostic determinants (gestational age, respiratory distress syndrome, multiple neonates, low birth weight, and kangaroo mother care) is 92.7% (95% CI: 89.9–95.4%). This prediction model was particular (specificity = 95%) in predicting preterm death, with a true positive rate (sensitivity) of 77%. The best cut point value for predicting a high or low risk of preterm death (Youden index) was 0.3 (30%). Positive and negative predictive values at the Youden index threshold value were 85.4 percent and 93.3 percent, respectively.

**Conclusion:**

This risk prediction model provides a straightforward nomogram tool for predicting the death of preterm newborns. Following the preterm neonates critically based on the model has the highest cost-benefit ratio.

## Introduction

Preterm birth is defined by the World Health Organization as any birth occurring before 37 weeks of pregnancy or within 259 days of a woman’s last regular menstrual period (LMP) (1). An estimated 15 million babies are born each year prematurely (2).

Evidence from different studies showed that birth weight, gestational age, marital status, required resuscitation after delivery, no antenatal care, plurality of pregnancy, sex of neonate, respiratory distress syndrome (RDS), hypothermia, not initiating exclusive breastfeeding, mode of delivery, non-cephalic presentation, fifth minute APGAR score < 7, did not receive kangaroo mother care (KMC) were risk factors in preterm mortality (3–9).

In 2020, over 6,500 newborn deaths occured every day, resulting in 2.4 million children dying in their 1st month of life (10, 11). Neonates mainly die due to preterm birth. Complications from preterm birth are the leading cause of death in children under five. Preterm complications account for 35 percent of total newborn fatalities in 2017, according to WHO and the Maternal and Child Epidemiology Estimation Group estimates (12). Each year, almost one million neonates and children under five die due to preterm birth problems (13, 14). Compared to Europeans, preterm newborns had 12 times higher risk of death in Africa. Preterm birth is the second most significant cause of under-five death in Sub-Saharan Africa, accounting for 12.1 percent, while preterm or low birth weight accounts for more than half of newborn mortality in East Africa (15). Preterm birth and associated complications account for more than one-fifth of newborn death in Ethiopia (15–20).

Predictive models are used to help clinicians determine if a clinical disease exists (diagnostic models) or whether an event will occur within the time frame (prognostic models) to aid in decision-making (21). Risk assessment and outcome prediction are critical tools (22). Predictions can be utilized in the prognostic context to construct therapeutic decisions based on the risk of developing a given result or event within a specific time frame (23). Predictions made by clinicians without using prediction tools are highly unreliable in the care of premature infants.

Continuous positive airway pressure (CPAP), surfactant administration for newborns with RDS, and phototherapy for jaundiced neonates are among the WHO recommendations from 2015 to increase the chances of survival and health outcomes for preterm infants. However, most of the above-mentioned preterm care facilities, such as radiant warmers, CPAP, surfactant, mechanical ventilators, and phototherapy, are sparse or non-existent in most of Ethiopia’s public hospitals. As a result, developing predicted probability models to forecast the mortality of preterm neonates could be beneficial, especially in low-resource settings with limited resources. Predictive models developed in developed countries might not be suitable for developing countries because they vary with the use of Biomarkers, sophisticated facilities, and devices that are not readily accessible due to resource or practical limitations in low-resource settings.

A predictive model allows real-time preterm neonates risk stratification, which guides primary attention to care for the good health outcome of the neonates. There is no study on the risk prediction of preterm neonatal mortality in Ethiopia. Identifying prognostic predictors of preterm mortality in the setting would be vital to decrease deaths of preterm mortality and meet sustainable development goal (SDG) targets on time. According to the Lancet report, if all nations achieved the SDG newborn mortality target by 2030, more than 5 million deaths may be averted over the next 10 years (24). Two-thirds of Sub-Saharan African countries, on the other hand, are on the verge of missing the SDG newborn death target. More than half of these nations must triple their current annual rate of reduction (ARR) for newborn mortality to accomplish the SDG target on time, and another 14 countries must at least double their current ARR. Ethiopia is one of the countries that will need to step up its efforts and accelerate progress to meet the SDG target if it is to meet it by 2030 (10, 11, 14). Therefore, this study tries to develop a nomogram for clinical risk prediction of preterm neonate death in Ethiopia, 2021.

## Materials and Methods

### Study Design, Area, and Period

A prospective institutional-based observational study design was employed in South Gondar zone public hospital, Northwest Ethiopia. Among eight public hospitals, this study was done in Addis- Zemen primary hospital, Wegeda primary hospital, Nefas Mewcha primary hospital, and Debretabor General Hospital. The study was conducted from December 2020 to May 2021.

### Eligibility Identification

All preterm neonates delivered and admitted in South Gondar Zone Public Hospitals were a source population. Preterm neonates who were delivered in the labor ward and admitted to NICU within 72 h of life were included in the study. Pregnant women without reliable last menstrual period or pregnant women without an early ultrasound report (first tri -minister), incomplete prenatal, delivery, and neonatal chart, and preterm neonates without parents were excluded during the data collection period.

### Sample Size Determination and Sampling Procedure

Since there is no prior prediction study for preterm neonates’ death in a similar setting to calculate the sample size, as the rule of thumb, ten events/preterm neonate death/per covariates were considered. Thirteen covariates were recruited in the final model from a total of 36 prognostic maternal and neonatal determinants based on literature or clinical experience. From a total of 456 neonates prospectively followed, there were 132 preterm neonate deaths that occurred.

Preterm neonates delivered from December 1, 2020 to May 30, 2021, delivered and admitted in a neonatal intensive care unit (NICU) in South Gondar zone public hospitals, were included. Eligible participants came up in the labor ward and admitted neonatal intensive care unit with consecutive sampling techniques. Proportional allocation based on the monthly average number of preterm delivered and admitted has been determined for each public hospital. We followed 204 preterm neonates at Debre Tabor Hospital, 76 preterm neonates at MekaneEyesus Hospital, 112 preterm neonates at Wegeda Hospital, and 28 preterm neonates at AdisZemen Hospital for 6 months during the data collection period.

### Measurement and Data Collection Procedure

We have used a reliable last menstrual period or an early ultrasound (first trimester) to determine gestational age. Data were collected based on an adapted interview-administered questionnaire and reviewing medical records. The questionnaire was adapted by reviewing different pieces of literature, including maternal socio-demographic, maternal medical, obstetric, prenatal, and neonatal variables, including age of the mother, the residence of the mother (urban, rural), marital status (union/non-union), pre-pregnancy body mass index, mother occupation, mother education, gestational age (late, moderate, very, extremely very preterm), parity, current pregnancy complications [preeclampsia/eclampsia/pregnancy-induced hypertension, preterm premature rupture of membrane, antepartum hemorrhage (APH), cord prolapse, and Rh factor], birth interval, antenatal care (ANC) follow-up, the plurality of the child (single, multiple), pregnancy intension (wanted/unwanted), mode of delivery, presentation, magnesium given for this pregnancy, antenatal steroids given or not, sex of the neonate, birth weight, age of the neonate, any resuscitation during delivery, sepsis, perinatal asphyxia (PNA), feeding status, APGAR score, kangaroo mother care (KMC) given or not, type of preterm (spontaneous or induced), respiratory distress syndrome (RDS), hypothermia, jaundice, congenital anomaly, and hypoglycemia.

The data were collected by 12 BSc neonatal nurses and four assistant lecturers in nursing supervisors. Trained data collectors interviewed mothers who were at 28 to less than 37 weeks (36 + 6 days) gestation at the labor ward and were abstracted pregnancy, delivery, and infant data from the chart and followed during a 24-h period until discharge, transfer, death, or 42 postmenstrual ages after birth, and their outcome was recorded.

Infant death between birth and 42 postmenstrual ages was declared dead. Neonates beyond 42 postmenstrual ages, referred to, and left with medical advice, were declared alive.

Neonatal morbidity was immediate neonatal complications, including (APGAR score, RDS, hyperbilirubinemia, and sepsis of preterm infants within 42 PMAs after birth (7). Sepsis is defined as clinical sign symptoms with risk factors in lab tests (microscopic) greater than two hematologic criteria (25). “Neonatal respiratory distress was diagnosed with the presence of one or more symptoms of tachypnea, intercostal muscle retraction, grunting, nasal flaring, and cyanosis” (26). Birth asphyxia is defined as “the failure to initiate and sustain breathing at birth.”

### Data Quality Control

Questionnaires were pretested with a 5% sample size at Mekane Eyesus primary hospital. The training was given for data collectors and supervisors for 1 day before and 1 day after the pretest regarding the study’s objective, data collection tool, and ways of data collection and checking the completeness of the data collection tool.

### Data Management and Analysis

Epi-Data version 4.6 software was used to enter the data. R-programming version 4.0.5 was used to analyze the data. In the beginning, variable selection in the multivariable model was made using least absolute shrinkage and selection operator (LASSO) regression. Two-sided *P*-values of less than 0.05 were considered statistically significant.

Model calibration was assessed by plotting declines of the predicted probability of preterm death in each decline and fitting a smooth line. The model discrimination probability was checked by using the area under ROC (AUROC) curve. “givitiR” R-packages were used to check the calibration plot. Bi-normal smoothing bootstrapping technique was used to validate AUROC internally. After bootstrapping, the model’s predictive performance was considered the performance that can be expected when the model is applied to future similar populations. Decision curve analysis (DCA) was used to evaluate the model’s clinical and public health impact.

The Youden index value, which classified high or low risk, was used to predict the likelihood of preterm death. Sensitivity, specificity, and the positive and negative predictive values were computed. The probability of preterm death for each neonate was predicted using the linear predictor of estimated risk of death, which is: P (Risk score for each patient) = risk score X prognostic determinant + … + N (risk score) X N (prognostic determinant). Additionally, a nomogram graphical presentation was prepared to construct an easily clinically applicable individualized prediction of preterm neonate risk of death.

### Ethical Considerations

The study participants were informed about the purpose, risk, benefits, and confidentiality of the study, and informed and voluntarily signed written consent has been obtained during the interview previously. Ethical approval was obtained from Debre Tabor University, College of Health Sciences, and the Institutional Review Board (IRB).

## Results

### Maternal Socio-Demographic Characteristics

A total of 456 preterm neonates were involved in this study. The median age of the respondents was 26 years. Most of the respondents (95.6%) were married, followed by single status (3.5%). Thirty-eight percent of the respondents were unable to read and write. Regarding the occupation of the respondents, 364 (79.8%) of them were housewives, and 68 (14.9%) of them were employees. Three hundred (65.8%) of the respondents were rural residents (Table 1).

**TABLE 1:** Maternal socio-demographic characteristics in south Gondar zone public hospitals, northwest Ethiopia, 2021.

Variable	Category	Frequency	Percent
Marital status	Single	16	3.5
	Married	436	95.6
	Divorce/windowed	4	0.9
Educational status	Unable to read and write	177	38.8
	Read and write	17	3.7
	Primary(1–8)	166	36.4
	Secondary(9–12)	40	8.8
	College and above	56	12.3
Occupation	Housewife	364	79.8
	Employed	68	14.9
	Merchant	12	2.6
	Student	12	2.6
Residence	Urban	156	34.2
	Rural	300	65.8
Pre-pregnancy BMI	Less than 18.5	88	19.3
	18.5–24	220	48.2
	Greater than 24	148	32.5

### Maternal Obstetric-Related Characteristics

Of the total respondents, one hundred seventy-two (37.7%) were Para-one. Most of the mothers (97.4%) have ANC follow-up during this pregnancy. Four hundred sixteen (93.7%) have four times and above ANC follow-up among those with ANC follow-up. About the delivery route, the majority (91.2%) of mothers had vaginal route delivery. Seventy-six (16.2%) of the mothers encountered complications during this pregnancy. APH was the most common one 24 (31.6%) among those who had complications during this pregnancy. The median afterbirth interval of the respondents was 36 months for those gravid two and above (Table 2).

**TABLE 2:** Maternal obstetric-related characteristics in south Gondar zone public hospitals, northwest Ethiopia, 2021.

Variables	Category	Frequency	Percent
Parity	One	172	37.7
	Greater than one	284	62.3
ANC	Yes	444	97.4
	No	12	2.6
No of ANC	Two	4	0.9
	Three	24	5.4
	Four and above	416	93.7
Route of delivery	Vaginal route	416	91.2
	Cesarean section	40	8.8
The plurality of the neonate	Singleton	336	73.7
	Multiple	120	26.3
Presentation	Cephalic	440	96.5
	con –Cephalic	16	3.5
Complication during this Pregnancy	Yes	76	16.7
	No	380	83.3
Type of complication	RH negative	4	5.2
	APH	24	31.6
	PIH	16	21.1
	Prolonged/Obstructed	8	10.4
	Urinary tract infection	4	5.2
	Cord Prolapse	4	5.2
	Premature rapture of membrane(PROM)	12	15.6
	Maternal fever	4	5.2
Antibiotics given in this Admission for the index pregnancy	Yes	24	5.3
	No	432	94.7
Pregnancy intention	Wanted	452	99.1
	Unwanted	4	0.9

### Neonatal-Related Characteristics

One hundred eighty-four (40.4%) of neonates were with gestational age between 32 and 34 weeks. Two hundred forty (52.6%) of the neonates were male (Table 3).

**TABLE 3:** Neonatal-related characteristics among preterm neonates in south Gondar zone public hospitals, northwest Ethiopia, 2021.

Variables	Category	Frequency	Percent
Gestational age	Less than 32 weeks	76	16.7
	32–34 weeks	196	43.0
	Greater than 34 Weeks	184	40.4
Any resuscitation at delivery	Yes	36	7.9
	No	420	92.1
Sex	Male	240	52.6
	Female	216	47.4
Type of preterm	Spontaneous	448	98.2
	Induced	8	1.8
Type of feeding	Breast milk	252	55.3
	Express breastfeeding by nasogastric tube	108	23.7
	Formula milk	4	0.9
	Nothing per mouth	92	20.2
Gestational age for weight	Appropriate for gestational age	440	96.5
	Small for gestational age	16	3.5
KMC	Yes	110	24.1
	No	346	75.9

### Immediate Morbidity of Preterm Neonates

One-fourth of the neonates had RDS. The majority (89.9%) of preterm neonates were hypothermic during admission. Thirty-six percent of preterm neonates were diagnosed with sepsis. Forty-four (9.6%) preterm neonates had PNA. Seven percent (27) of the preterm neonates had hypoglycemia (Table 4).

**TABLE 4:** Immediate morbidity among preterm neonates in south Gondar zone public hospitals, North West Ethiopia, 2021.

Morbidities	Category	Frequency	Percent
RDS	Yes	116	25.4
	No	340	74.6
Hypothermia	Yes	410	89.9
	No	46	10.1
Sepsis	Yes	164	36.0
	No	292	64.0
PNA	Yes	44	9.6
	No	412	90.4
Jaundice	Yes	56	12.3
	No	400	87.7
Congenital anomaly	Yes	8	1.7
	No	448	98.3
Hypoglycemia	Yes	36	7.9
	No	420	92.1

### A Predictive Model for Preterm Neonates Death

The maternal socio-demographic, obstetric, and neonatal-related prognostic determinants were considered to predict preterm mortality. In the final model, the plurality of the neonate (multiple) has no RDS, gestational age of less than 32 weeks, low birth weight, and KMC were the remaining significant predictors of preterm mortality (Table 5).

**TABLE 5:** Multivariate binary logistic regression results among preterm neonates in south Gondar zone public hospitals, North West Ethiopia, 2021.

Variables	Category	Death		COR (95% CI)	AOR (95% CI)
		Yes	No		
Marital status	Union	120	316	1	1
	Not union	12	8	4 (1.58, 9.90)	1.13 (0.22, 5.91)
The plurality of the neonate	Singleton	76	260	1	
	Multiple	56	64	2.99 (1.93, 4.65)	**2.6 (1.19, 5.58)**
Presentation	Cephalic	124	316	1	
	Non-cephalic	8	8	2.55 (0.94, 6.94)	4.4 (0.63, 31.34)
Neonate Sex	Male	92	148	1	
	Female	40	176	0.37 (0.24, 0.56)	1.3 (0.59, 2.69)
RDS	Yes	96	20	1	**1**
	No	36	304	0.02 (0.014, 0.05)	**0.02 (0.01,0.04)**
Antenatal corticosteroid given	Yes	6	28	1	1
	No	126	296	1.99 (0.80, 4.92)	0.40 (0.11, 1.52)
GA	Less than 32 weeks	52	24	1	**16.3 (6.04, 44.14)**
	32–36 + 6 weeks	80	300	0.12 (0.07,0.21)	**1**
Hypothermia	Yes	126	284	2.96 (1.22, 7.15)	1.8 (0.53, 6.15)
	No	6	40	1	**1**
PNA	Yes	28	16	1	1
	No	104	308	0.19 (0.10,0.37)	0.52 (0.17, 1.62)
Sepsis	Yes	68	96	1	1
	No	64	228	0.4 (0.26, 0.60)	0.60 (0.26, 1.37)
Birth weight	≥2500	20	117	1	**1**
	Less than 2500	112	207	3.17 (1.87, 5.36)	**4.1 (1.70, 9.78)**
KMC given	Yes	9	101	1	**1**
	No	123	223	6.19 (3.02, 12.67)	**10.1 (3.36, 30.7)**
Route of delivery	Vaginal	292	124	1	**1**
	Cesarean	32	8	0.589 (0.26,1.733)	0.5. (0.21,1.2)

*Bold = significant.*

After the multivariable logistic model, a total of five prognostic determinants were left for the prediction of preterm mortality, and the relative contribution of each prognostic determinant to the probability of preterm mortality was calculated by dividing each beta coefficient by the lowest beta coefficient and rounding to the nearest integer (score chart rule formula) (Table 6).

**TABLE 6:** Prognostic determinants in south Gondar zone public hospitals, northwest Ethiopia, 2021.

Prognostic determinants	AOR (95% CI)	Regression coefficient	Contribution to risk score
The plurality of the neonate (multiple)	2.6 (1.19, 5.58)*	0.77	1
Has no RDS	0.02 (0.01, 0.04)***	−4.4	6
Gestational age less than 32 weeks	16.3 (6.04, 44.14)***	2.66	4
Low birth weight	4.1 (1.70, 9.78)***	1.52	2
Kangaroo mother care is not given	10.1 (3.36, 30.7)***	2.1	3
Constant−**1.54**			

N.B.: Significant codes: ***< 0.001, * <0.05.

Therefore, the probability of preterm mortality among preterm neonates using the **linear** prediction formula was:

**Linear predictor of the model (l p)** = −1.54 + 2.66 * gestational age less than 32 weeks + 0.77 *multiple pregnancy- 4.4; *has no RDS, + 2.1; *has not gotten KMC, + 1.52; *low birth weight.

### Model Discrimination Probability and Calibration Plot

**FIGURE 1:**
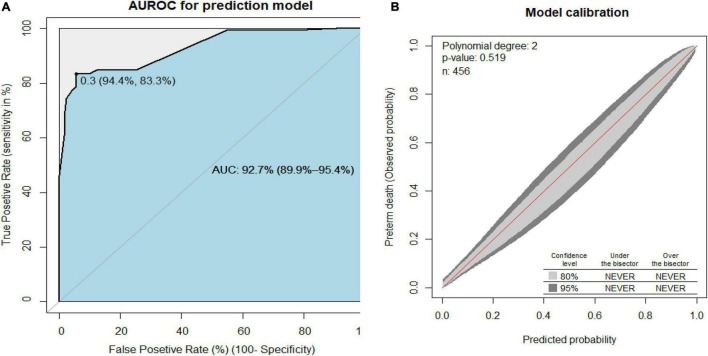
**(A)** Area under the receiver operating characteristics curve (AUROC) for the reduced prediction model and **(B)** the prediction model calibration plot to predict preterm birth neonates.

The area under the receiver operating characteristics curve (AUROC) had a discrimination power of 92.7% (95% CI: 89.9–95.4) (Figure 1A). The final prediction model was good calibrated (p-value = 0.519) (Figure 1B). The prediction model was particular (specificity = 95%) in its ability to predict preterm death, with a true positive rate (sensitivity) of 77%. This prediction had a low false-positive rate of 4% among the actual death of the preterm neonates. The model prediction accuracy was 90% (Supplementary File 1). The prediction model was internally validated using 2,000 bootstrap replicates.

### Model Cost-Benefit Analysis (Decision Curve Analysis)

**FIGURE 2:**
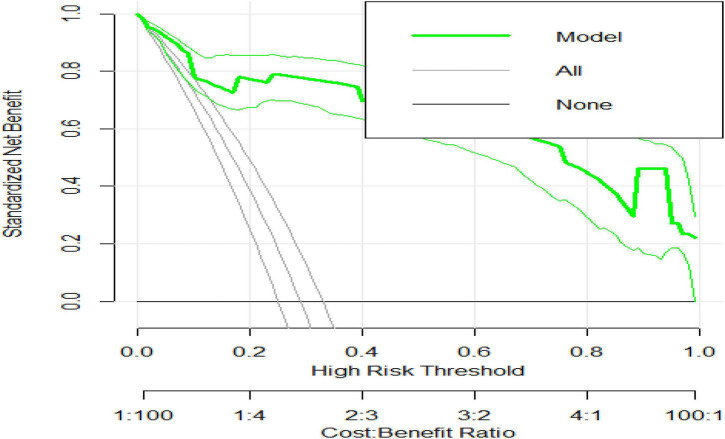
Decision curve analysis for prediction of preterm neonates’ death.

The cost-benefit analysis of the prediction model was compared between no follow-up for all the preterm neonates, followed by all the preterm neonates, and critically follow selectively based on these predictors. For example, if we critically follow all preterm neonates at a threshold risk of death probability of 30%, we will find 30% prevalence of preterm death. Whereas, if we follow the preterm neonates based on the prediction model, the preterm neonates need critical follow-up at the risk of preterm death threshold of more than 80%. Therefore, following the preterm neonates selected based on the model has the highest cost-benefit ratio (Figure 2).

### Nomogram for Prediction of Preterm Death

**FIGURE 3:**
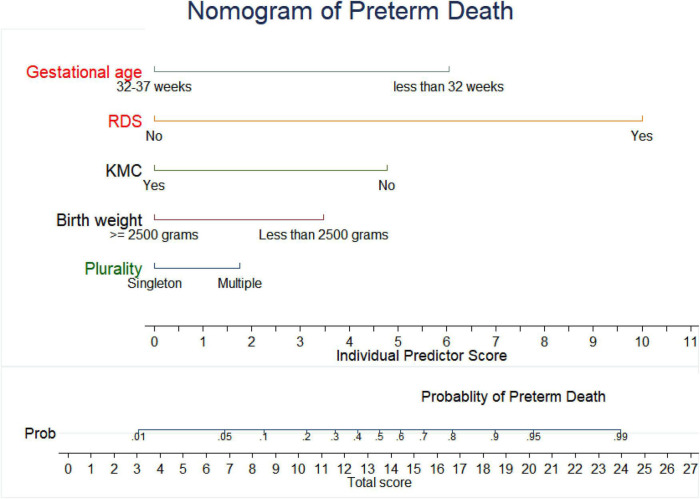
Nomogram of a prognostic model to estimate the probability of preterm death.

To construct an easily clinically applicable individualized prediction of preterm neonate risk of death was prepared in nomogram graphical presentation (Figure 3). The best threshold (Youden index) cut point value was 0.3 (30%) to predict a high or low risk of preterm death (Figure 1A). The maximum sensitivity and specificity of the Youden index cut point value were 83.3 and 94.4%, respectively (Figure 1A). The positive predictive and negative predictive values at the Youden index threshold value were 85.4 and 93.3%, respectively. The linear predictor of five parameters is estimated in the probability of preterm death in the nomogram to predict the preterm neonate death. For example, if a preterm neonate was born with multiple births, low birth weight, and the preterm neonate was not under KMC, the predicted probability of preterm death is calculated as follows. The total point for the three predictors was the sum of each predictor point, which was 1.8 + 3.5 + 4.8 = **10.1.** Therefore, the probability of preterm death with the corresponding total points in the nomogram was 0.2 (20%), indicating a low risk of preterm death. Whereas a preterm neonate had RDS and low birth weight, the total points were 10 + 4.8 = 14.8. Thus, the probability of preterm death, approximately 0.68 (68%), is declared a high risk of preterm death (Figure 3).

## Discussion

In our study, a combination of five sets of prognostic determinants (gestational age, RDS, multiple neonates, low birth weight, and KMC) results in the discernment power of area under the receiver operating curve (AUROC), 92.7% (95% CI: 89.9–95.4%). Having an e in the model’s AUROC of 0.93 indicates that it is 93 percent accurate incapable of distinguishing between preterm neonates who died and those who survived. The higher the AUC indicates, the better the model predicts dead preterm neonates’ classes as death and alive preterm neonates’ classes as alive The area under the ROC curve (AUC) results was reflected excellent for AUC values between 0.9 and 1 (28, 29). Because it had an outstanding measure of separability of dying from alive preterm neonates, it is of paramount importance if the physician employs this tool to anticipate preterm neonates’ mortality. Following the preterm neonates selectively based on the model’s tool has the highest cost-benefit ratio by considering the false-positive rate.

Clinicians can determine the likelihood of preterm neonate risk of mortality by adding each value of the prognostic factors that the neonate possesses from the nomogram. This study’s nomogram graphical display offers a clinically useful customized prediction of preterm neonate death risk. The prognostic predictor of five factors was assessed in the probability of preterm death in the nomogram to predict preterm neonate death.

In this study, the maximum sensitivity and specificity of the Youden index cut point value were 83.3 and 94.4%, respectively. It means that this tool has an 83% probability of correctly identifying all those who do, indeed, have died from among preterm neonates who have died and the probability of avoiding neonates who have not died when they have. Similarly, this model has a 94% probability of correctly identifying; from among preterm neonates who do not die, all those who do, indeed, have not died and not categorizing some neonates as died when, in fact, they do not die (30). These findings suggest that this prediction had a low false-positive rate among actual preterm neonatal deaths, indicating an essential role in predicting preterm birth.

At the Youden index threshold value, the positive and negative predictive values were 85.4 and 93.3%, respectively. This indicates that this tool has an 85 percent chance of correctly identifying all newborns that have died from among preterm neonates who may or may not have died and not categorizing some neonates as dead while they are alive. Furthermore, this tool has a 93 percent chance of correctly identifying all neonates who do not die among preterm neonates who may or may not die while correctly categorizing some neonates as alive when they are not. It accurately predicted that the neonate perished and, if true, projected that the neonate was alive. It has good predicted the neonate died and if was died and predicted the neonate was alive if was alive. As a result, it can accurately predict preterm mortality.

In this study, RDS is one prognostic determinant for preterm mortality. It is similar to different studies on neonatal mortality prediction (31, 32). The possible justification might be the lack of surfactant in premature newborns, which raises the surface tension inside the small airways and alveoli, limiting the immature lung’s compliance and causing hypoxemia and lactic acidosis. In addition to a lack of surfactant, the preterm infant’s immature lung has lower compliance, decreased fluid clearance, and immature vascular development, all of which predispose the lung to damage and inflammation, complicating the normal development of alveoli and pulmonary vasculature (33). This may further complicate and increase neonatal mortality.

Gestational age less than 32 weeks was another prognostic determinant for preterm mortality. It is consistent with different studies on the prediction of neonatal mortality (31, 32). The possible justification might be that better physiological and immunological maturity occurs as gestational age increases.

Multiple neonates are one prognostic determinant for preterm mortality. It is similar to a study done in Ohio that revealed that twin pregnancy was predicted risk of adverse pregnancy outcomes, including neonatal death (34). It might be due to twins being more likely LBW because of restricted growth and increased rates of obstetric complications, such as premature separation of the placenta and had perinatal neonatal complications. Multiple births are high-risk pregnancy and birth (35, 36).

Low birth weight is one prognostic determinant for preterm mortality. This might be lower birth weight (16) prone the neonate to metabolic disorders and other complications that increase the risk of death. It is consistent with different studies on premature infants (33, 37) and among both term and preterm neonates (38, 39).

Kangaroo Mother Care is one prognostic determinant for preterm mortality. One reason is that KMC prevents hypothermia, which causes neonatal mortality (27, 32). KMC offers many more benefits besides thermal protection, notably a successful initiation and maintenance of lactation and preventing apnea of prematurity. So it averted the death of preterm neonates. KMC markedly reduces neonatal mortality among preterm babies and significantly decreases severe morbidity, especially from infection (37, 38).

## Conclusion

In our study, a combination of five sets of prognostic determinants (gestational age, RDS, multiple neonates, low birth weight, and KMC) was estimated in the probability of preterm death in the nomogram. This nomogram can be used to graphically predict the risk of death among preterm neonates. In settings with similar demographics, the risk prediction tool can assist clinicians and health care providers to predict preterm neonates’ death and target interventions. Following the preterm neonates based on the selectively using the model has the highest cost-benefit ratio.

## Data Availability Statement

The original contributions presented in the study are included in the article/Supplementary Material, further inquiries can be directed to the corresponding author.

## Ethics Statement

The studies involving human participants were reviewed and approved by the Debre Tabor University, College of Health Sciences, and Institutional Review Board (IRB). Written informed consent to participate in this study was provided by the participants’ legal guardian/next of kin.

## Author Contributions

HH was involved in conceptualization, methodology, data cleaning, data analysis, interpretation, drafting, reviewing, and revising of the manuscript. ST was involved in methodology, data cleaning, data analysis, interpretation, drafting, reviewing, and revising of the manuscript. Both authors read and approved the final manuscript.

## Conflict of Interest

The authors declare that the research was conducted in the absence of any commercial or financial relationships that could be construed as a potential conflict of interest.

## Publisher’s Note

All claims expressed in this article are solely those of the authors and do not necessarily represent those of their affiliated organizations, or those of the publisher, the editors and the reviewers. Any product that may be evaluated in this article, or claim that may be made by its manufacturer, is not guaranteed or endorsed by the publisher.
