# Supplementary material for: Development of a Nomogram for Clinical Risk Prediction of Preterm Neonate Death in Ethiopia
Source: Front Pediatr. 2022 May 27;10:877200. doi: 10.3389/fped.2022.877200 (PMC9184443; doi:10.3389/fped.2022.877200)
Supplement: Supplementary file 1 [file Table_1.DOCX]

**Supplementary file 1. The sensitivity, specificity, Positive predictive value, and negative predictive value of the prediction model.**

|  | | Actual value (preterm death) | | Total |
| --- | --- | --- | --- | --- |
|  |  | Yes | No |  |
| Prediction model | Yes | TP = 102 | FP = 14 | 116 |
|  | No | FN = 30 | TN = 310 | 340 |
| Total | | 132 | 324 | 456 |

**Therefore,**

Sensitivity $=\frac{TP}{TP + FN} = \frac{102}{102 + 30} =77.3\%$ , Specificity $=\frac{TN}{TN +FP} = \frac{310}{310 + 14} =95.7\%$

False positive rate $=\frac{False \mathrm{positives}}{Negatives} = \frac{14}{324} =4.3\%$

Accuracy of prediction model $=\frac{Correct prediction}{Test size (Sample size)} = \frac{TP+TN}{Test size (Sample size)} \frac{412}{324456} =90.4\%$
